# The Impact of Smoking in Adolescence on Early Adult Anxiety Symptoms and the Relationship between Infant Vulnerability Factors for Anxiety and Early Adult Anxiety Symptoms: The TOPP Study

**DOI:** 10.1371/journal.pone.0063252

**Published:** 2013-05-16

**Authors:** Steven Moylan, Kristin Gustavson, Evalill Karevold, Simon Øverland, Felice N. Jacka, Julie A. Pasco, Michael Berk

**Affiliations:** 1 School of Medicine, Deakin University, Geelong, Victoria, Australia; 2 Division of Mental Health, Norwegian Institute of Public Health, Oslo, Norway; 3 Faculty of Psychology, University of Bergen, Bergen, Norway; 4 NorthWest Academic Centre, Department of Medicine, The University of Melbourne, St. Albans, Victoria, Australia; 5 Orygen Youth Health Research Centre, Centre for Youth Mental Health, Parkville, Victoria, Australia; 6 The Florey Institute for Neuroscience and Mental Health, Parkville, Victoria, Australia; 7 Department of Psychiatry, Melbourne University, Parkville, Victoria, Australia; 8 Barwon Health, Geelong, Victoria, Australia; Institute of Psychiatry at the Federal University of Rio de Janeiro, Brazil

## Abstract

Cigarette smoking is increased in people with trait anxiety and anxiety disorders, however no longitudinal data exist illuminating whether smoking in adolescence can influence the developmental trajectory of anxiety symptoms from early vulnerability in infancy to adult anxiety expression. Using *The Tracing Opportunities and Problems in Childhood and Adolescence (TOPP) Study*, a community-based cohort of children and adolescents from Norway who were observed from the age of 18months to age 18–19years, we explored the relationship between adolescent smoking, early vulnerability for anxiety in infancy (e.g. shyness, internalizing behaviors, emotional temperaments) and reported early adult anxiety.

Structural equation modeling demonstrated that adolescent active smoking was positively associated with increased early adulthood anxiety (β = 0.17, p<0.05), after controlling for maternal education (proxy for socioeconomic status). Adolescent anxiety did not predict early adult smoking. Adolescent active smoking was a significant effect modifier in the relationship between some infant vulnerability factors and later anxiety; smoking during adolescence moderated the relationship between infant internalizing behaviors (total sample: active smokers: β = 0.85,p<0.01, non-active smokers: ns) and highly emotional temperament (total sample: active smokers: β = 0.55,p<0.01,non-active smokers: ns), but not shyness, and anxiety in early adulthood. The results support a model where smoking acts as an exogenous risk factor in the development of anxiety, and smoking may alter the developmental trajectory of anxiety from infant vulnerability to early adult anxiety symptom expression. Although alternative non-mutually exclusive models may explain these findings, the results suggest that adolescent smoking may be a risk factor for adult anxiety, potentially by influencing anxiety developmental trajectories. Given the known adverse health effects of cigarette smoking and significant health burden imposed by anxiety disorders, this study supports the importance of smoking prevention and cessation programs targeting children and adolescence.

## Introduction

Multiple population based epidemiological studies, utilizing cross-sectional and prospective designs, have demonstrated higher rates of cigarette smoking in individuals with anxiety disorders [1]–[3] and increased anxiety symptoms [4](*see review, Moylan et al* [5]). These increased rates of smoking are quite startling. For example, data from the US National Comorbidity Study revealed rates of current smoking rates between 38–46% in those with an anxiety disorder compared to 22.5% in those with no mental disorder [1]. Rates of smoking amongst those with anxiety disorders in New Zealand were estimated at approximately 30% (compared to 20% in those without a mental disorder) in a nationally representative study, with those individuals consuming approximately 16% of all cigarettes in New Zealand [3]. Despite significant investigation [4], [6]–[13] a complete understanding of this association remains elusive. Such understanding is important, as it could inform interventions aimed at reducing the morbidity associated with this association. Three non-mutually exclusive models could explain this association [5].

First, the observed association could be explained by shared vulnerability, where biological and/or environmental factors increase the likelihood of smoking and of developing increased anxiety [14], [15]. As an example, lower socioeconomic status, which is associated with multiple disease risk factors and mental disorder expression, is associated with both increased smoking [16], [17] and anxiety [18]. Second, anxiety may increase smoking-related behaviors [4], [19]–[23]. Evidence suggests individuals with increased anxiety do display increased smoking behaviors [4], [19]–[23], with various factors, including an increased vulnerability to start smoking in response to peer pressure [4], [16] and use of cigarettes for anxiety-relief, proposed as possible explanations. Finally, smoking may predispose to increased anxiety levels through a variety of mechanisms [20], [21], [24]–[27]. For example, smoking adversely impacts respiratory health and autonomic control; effects that potentially increase vulnerability for developing anxiety [13], [28]–[31]. Furthermore, some literature suggests a direct effect of cigarette smoke and nicotine on normal neurodevelopment [32], [33], which may increase the chance of developing anxiety in later life. In support, rats exposed to nicotine in early life exhibit more anxiety-like states in adulthood than those exposed at a later age [34].

It is possible that models proposed to explain the adverse effects of cigarette smoke exposure on development of numerous medical disorders may be relevant to anxiety development. Exposure to cigarette smoke in childhood and adolescence is known to increase adult disease expression and severity of multiple physical illnesses [35], including aortic atherosclerosis [36] and asthma [37], [38], particularly in those with a pre-existing disease vulnerability (e.g. family history). In these disorders, cigarette smoke may act as an exogenous risk factor that compounds pre-existing vulnerability to intensify disease development and severity. A similar pathogenic model could be relevant for mental disorders: commencing smoking at an earlier age, particularly during key stages of neurodevelopment such as adolescence, may exacerbate an underlying vulnerability for anxiety. In this model, exposure cigarette smoke could alter the developmental trajectory of anxiety through effects on underlying neurobiology [39] and leave individuals more susceptible to increased anxiety in the long term. These effects could result from direct effects of cigarette smoke components (e.g. nicotine and free radicals) leading to disturbance in the function and regulation of neurotransmitter systems (e.g. the cholinergic system) and/or by direct cellular damage secondary to increased oxidative and nitrosative stress. This model is supported by recent observations that early onset smoking is associated with shorter time to onset of anxiety disorders [40].

Some early childhood behaviors are predictive of increased adult anxiety, including internalizing behaviors, shyness and a highly emotional temperament [41]–[43]. These behaviors may be conceptualized as early markers of an underlying risk for increased adult anxiety. Through testing whether or not exposure to cigarette smoke moderates the developmental trajectory of anxiety from these early observed anxiety behaviors to adult anxiety expression, we have the opportunity to test whether a neurodevelopmental model could apply to anxiety development. Such insights could provide a basis for future endeavors in further understanding anxiety pathogenesis, and how cigarette smoke influences this process.

This study uses a large longitudinal cohort, the *Tracing Opportunities and Problems in Childhood and Adolescence (TOPP) Study*, to assess the prospective relationship between adolescent smoking and adult anxiety symptoms. The study tests two hypotheses: that adolescent smoking is associated with increased early adult anxiety symptoms, and that adolescent smoking moderates the relationship between infant vulnerability for anxiety and early adult anxiety.

## Methods

### Participants and Study Procedures

We used data from the TOPP Study [44], a prospective community-based observational study of children and adolescents from eastern Norway. In 1993, 929 families attending 18-month vaccination visits from 19 health care areas in eastern Norway completed the initial survey. The 19 health care areas were representative of the diversity of Norwegian social environments. The sample was predominantly middle class ethic Norwegian families, 28% of whom resided in large cities, 55% in densely populated areas and 17% rurally. Mothers were on average 30 years of age (SD = 4.7; Range: 19–46years) at baseline assessment. Data collected from child health clinics demonstrated no association between maternal age, employment status, education, marital status of number of children and likelihood of study participation. The participating families were subsequently invited to undertake further questionnaires when their children were 2–3 years, 4–5 years, 8–9 years, 12–13 years, 14–15 years, 16–17 years and 18–19 years. The parents provided all information until the age of 8–9 years, after which information was collected from parents and children. All participants provided informed consent and the study procedures were approved by the Norwegian Ethical Committee.

At baseline, age 18months, information was available from 913 mothers on their infants (49% male), with 456 (50%) adolescent respondents at age 14–15 and 441 (48%) adolescent respondents at ages 18–19 years. Multiple baseline variables were tested to assess their association with study attrition at early adulthood follow up (age 18–19 yrs). After controlling for multiple-significance testing via Bonferroni correction, study attrition was only significantly related to maternal education (increased education associated with decreased drop-out) and gender (girls with less likelihood of drop-out). Hence gender and maternal education measured at baseline were included and controlled for in the models.

Basic descriptive statistics for independent and dependent variables are displayed in Table 1. Baseline maternal education, utilized as a proxy for socioeconomic status, was assessed at baseline using eight categories ranging from “seven years of primary school or less” to “university level with four or more years”. During the period of schooling for some parents, Norway had some alternative schooling options available on a limited basis that represented items 7–8years schooling and 10 years schooling. These are the equivalent of primary schooling and hence we have collapsed these items down into a single category “Primary Schooling Only”. Maternal report of infant vulnerability factors for anxiety was also taken from the baseline questionnaire, and included levels of emotionality, shyness and internalizing behaviors. Shyness and emotionality were assessed by the Emotionality, Activity and Sociability Temperament Survey (EAS) [45] where each temperament trait was measured by five items rated on a 5-point scale (1 = not typical to 5 = very typical). Shyness refers to the tendency to display inhibited and awkward behaviors in new social situations, and emotionality refers to the tendency to become easily and intensely aroused. Due to ambiguity in translation, one item from emotionality scale (“The child tends to be somewhat emotional”) and the shyness scale (“ The child makes friends easily”) was deleted, as has been undertaken in other studies using this dataset [43]. Factor analytic investigation of the EAS has been undertaken in this data set, demonstrating good reliability (Emotionality: Cronbach α = 0.66; Shyness: Cronbach α = 0.75) and construct validity [46], [47]. Internalizing behaviors were assessed using two items (“Has many different worries, broods over things”, “Is often frightened by loud noises and unexpected things”) from the *Behaviour Checklist* [48], and one additional item pertaining to sadness (“Seems often, or for long periods, to be unhappy”). The items for each dimension were combined into a mean index.

**Table 1:** Descriptive statistics of included independent and dependent variables.

Baseline Variables	Frequency (%)	Anxiety Mean Scores	Frequency (%)
**Maternal Education**		**Anxiety - Age 14–15 yrs**	
≤7 years Schooling	7 (0.8%)	1–1.50	337 (73.9%)
Primary Schooling Only	85 (9.3%)	1.51–2.0	81 (17.8%)
1–2 years High Schooling	245 (26.9%)	2.01–2.5	29 (6.4%)
3 years High Schooling	231 (25.4%)	2.51–3.0	7 (1.5%)
≤4 years College/University	208 (22.9%)	3.01–3.5	2 (0.4%)
≥4 years College/University	134 (14.7%)	3.51–4.0	0 (0.0%)
**Shyness (mean score)**		**Anxiety - Age 16–17 yrs**	
1–2.00	339 (37.9%)	1–1.50	294 (79.0%)
2.01–3.00	444 (48.6%)	1.51–2.0	57 (15.3%)
3.01–4.00	116 (12.7%)	2.01–2.5	14 (3.8%)
4.01–5.00	15 (1.6%)	2.51–3.0	3 (0.8%)
		3.01–3.5	3 (0.8%)
**Emotionality (mean score)**		3.51–4.0	1 (0.2%)
1–2.00	207 (22.7%)		
2.01–3.00	489 (53.6%)	**Anxiety - Age 18–19 yrs**	
3.01–4.00	185 (20.3%)	1–1.50	351 (79.6%)
4.01–5.00	32 (3.5%)	1.51–2.0	56 (12.7%)
		2.01–2.5	20 (4.5%)
**Internalizing (mean score)**		2.51–3.0	11 (2.5%)
0–0.49	749 (81.9%)	3.01–3.5	2 (0.5%)
0.50–0.99	124 (13.6%)	3.51–4.0	1 (0.2%)
1.0–1.49	41 (4.5%)		
1.50–1.99	1 (0.1%)		

Smoking (child self-report) was assessed at ages 12–13 to 18–19 years. For ages 12–13 and 14–15, respondents endorsed a four-option categorical scale (“never smoked”, “have tried smoking”, “sometimes smoke” and “daily smoker”); for ages 16–17 and 18–19 a five-option categorical scale (“never smoked”, “have tried smoking”, “former smoker, now quit”, “sometimes smoke” and “daily smoker”) was used. Due to the limitation of quite small numbers of smokers in some categories, we decided to reclassify smokers into a dichotomous variable – “active smoking” versus “non-active smoking” – by collapsing categories 1–2 and 3–4 at ages 12–13 and 14–15, and categories 1–3 and 4–5 at ages 16–17 and 18–19. As only 1 respondent identified as an active smoker at age 12–13, our analysis focused on smoking at age 14–15.

Self-report anxiety was measured at ages 12–13 and 14–15 using items from the Generalized Anxiety Disorder Scale, a subscale of the Coolidge personality and neuropsychological inventory for children [49]. The scale measured twelve items drawn from DSM-IV [50] criteria for generalized anxiety disorder, separation anxiety disorder and social phobia on a four point continuous scale for anxiety symptoms experienced in the last two months. At ages 16–17 & 18–19, self-report anxiety was measured using 14 items relevant to anxiety in the Depression Anxiety Stress Scales – 42 (DASS42 [51]). DASS42 questions utilize a 0–3 scale, and we averaged these scores and added 1 to gain a score between 1–4 for each respondent. Increased anxiety scores equated to increased anxiety symptoms. This scale has shown good reliability, convergent and discriminant validity in non-clinical samples [52]. Additional information on validity and reliability of scales is available elsewhere [43], [46], [53].

To assist the reader, the results and discussion are presented such that age 18 months is defined as “infancy”, age 14–15 is defined as “adolescence” and age 18–19 is defined as “early adulthood”.

### Data Analytic Procedures

Statistical analyses were undertaken using SPSS version 17 and Mplus version 6.0 [54]. One smoker was an outlier in terms of anxiety score, and was recoded to level with the next highest anxiety score among the smokers. This procedure is in line with recommendations by Tabachnick & Fidell [55], who recommend recoding an outlier to a less extreme value.

A structural model was constructed in Mplus to examine how anxiety and smoking at age 14–15 were associated with smoking and anxiety at age 18–19. The WLSMV estimator was used due to the categorical nature of the smoking variable. The WLSMV estimator allows pairwise rather than listwise deletion and performs well under the MRAX missing data assumption (where missingness is associated with independent variables in the model) [56]. Maternal educational level was included in the models as a proxy for socioeconomic status [57]. We chose *a priori* to not control for depression. Due to the significant overlap between the self-reported anxiety and depressive symptoms, controlling for depression would have unacceptably increased the risk of a type 2 error. Given the low number of boys who reported active smoking at age 14–15 (n = 5), we only created models incorporating the total sample and girls only.

The moderating effect of smoking on the associations between childhood risk markers (temperamental emotionality, internalizing problems, and temperamental shyness) and anxiety at age 18–19 was then examined by running two-group analyses in Mplus. In these analyses the Robust Maximum Likelihood estimator was used, allowing use of all available information and accounting for the non-normal distribution of the measures. The associations between childhood risk markers and anxiety at age 18–19 were constrained to be equal among smokers and non-smokers and then allowed to be estimated freely in the two groups in a second step of these analyses. The fit of the unconstrained and constrained models were then compared to test for statistically significant differences. The smoking group was small in these analyses, and to reduce the ratio of estimated parameters/number of observations, the mean and variance of the maternal education variable was identified for the smokers first and then entered into the models.

We used adjusted chi-square difference values in difference testing of nested models as suggested by Satorra and Bentler [58]. However, in the model examining the moderating effect of smoking on the association between early internalizing problems and later anxiety, the adjusted chi-square difference turned out negative, and should thus not be interpreted [59]. The ML-estimator and regular chi-square difference was thus used in these analyses. An alternative to this methodology would have been to use the MLMV estimator and the diff-test option [59], but that would have required listwise deletion and hence a reduction in the already small active smoking group.

## Results

Between ages 14–15 years (“adolescence”) 21 (4.7%) adolescents (5 boys and 16 girls) self-reported active smoking. The mean anxiety score for the early adulthood group was 1·30 (SD 0.41). Between ages 18–19 (“early adulthood”), 85 (24.2%) adults (36 boys and 49 girls) reported active smoking and mean DASS anxiety score for the entire sample was 1.30 (SD 0.42). Active smoking during adolescence was correlated with active smoking in early adulthood (r = 0.3, p<0.001). In addition, anxiety scores during adolescence were correlated with anxiety scores during early adulthood (r = 0.4, p<0.001). Baseline information, smoking and anxiety scores across time points and correlations are displayed in Tables 1, 2, 3.

**Table 2:** Baseline smoking and anxiety scores and correlations.

	Anxiety (14–15)	Anxiety (16–17)	Anxiety (18–19)	Active Smoking (14–15)	Active Smoking (16–17)	Active Smoking (18–19)
**Mean (SD)/Frequency Yes (%) Total; Female**	0.33 (0.38); 0.38 (0.39)	0.30 (0.39); 0.40 (0.44)	0.30 (0.42); 0.37 (0.49)	21 (4.7%); 16 (76%)	45 (4.6%); 30 (67%)	85 (24.2%); 49 (58%)
**Anxiety 14–15**	x	r = 0.314**	r = 0.405**	r = 0.087	r = 0.035	r = 0.059
**Anxiety 16–17**	r = 0.314**	x	r = 0.586**	r = 0.042	r = 0.283**	r = 0.153*
**Anxiety 18–19**	r = 0.405**	r = 0.586**	x	r = 0.165**	r = 0.167**	r = 0.097
**Active Smoking 14–15**	r = 0.087	r = 0.042	r = 0.165**	x	r = 0.297**	r = 0.296**
**Active Smoking 16–17**	r = 0.035	r = 0.283**	r = 0.167**	r = 0.297**	x	r = 0.413**
**Active Smoking 18–19**	r = 0.059	r = 0.153*	r = 0.097	r = 0.296**	r = 0.413**	x
**Maternal Education (baseline)**	r = −.08	r = −.09	r = .00	r = −.07	r = −.05	r = −.11
**Emotionality (baseline)**	r = −0.101	r = 0.121	r = −0.075	r = −0.109	r = 0.058	r = −0.105
**Internalizing Behaviors (baseline)**	r = 0.065	r = −0.035	r = −0.079	r = 0.064	r = 0.001	r = −0.068
**Shyness (baseline)**	r = .12*	r = .17**	r = .08	r = .01	r = −.02	r = −.06

Baseline = 18 months of age,

* p<0.05,

** p<0.01.

**Table 3:** Frequency of smoking reported at different age categories.

Smoking Rates by Category	Frequency (%)
**Smoking Age 14–15**	
Never Smoked	336 (74.7%)
Have Tried Smoking	93 (20.7%)
Sometimes Smoke	13 (3.1%)
Daily Smoking	7 (1.6%)
**Smoking Age 16–17**	
Never Smoked	199 (53.6%)
Have Tried Smoking	119 (32.1%)
Former Smoker, Now Quit	8 (2.2%)
Sometimes Smoke	31 (8.4%)
Daily Smoking	14 (3.8%)
**Smoking Age 18–19**	
Never Smoked	130 (37%)
Have Tried Smoking	120 (34.2%)
Former Smoker, Now Quit	16 (4.6%)
Sometimes Smoke	58 (16.5%)
Daily Smoking	27 (7.7%

### Prospective relationship between adolescent smoking and early adult anxiety symptoms

**Figure 1:**
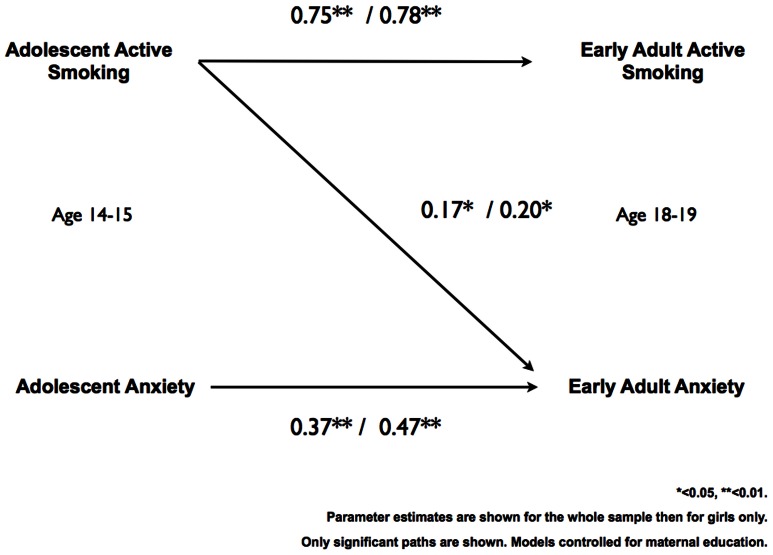
Structural model of associations between active smoking and anxiety scores in adolescence (age 14–15) and early adulthood (age 18–19). **Caption:** Adolescent active smoking at age 14–15 years in the total sample and for girls only was associated with increased anxiety symptoms scores at age 18–19 years (β coefficients displayed for total sample first, then for girls only). * p<0.05, ** p<0.01. The model is adjusted for maternal educational level. Only significant paths are shown. All paths were estimated in the model (df = 0). Model fit perfect (χ ^2^ = 0.00, RMSEA = 0.000, TLI and CFI = 1.00). The path from Adolescent active smoking to early adult active smoking is a probit regression coefficient. When translated into probabilities, the results show that at a mean level of maternal education and adolescent anxiety, active smoking during adolescence has 65% risk of being active smokers in early adulthood, while non-active smokers during adolescence has a 36% risk of active-smoking in early adulthood.

Structural Equation Modeling found no relationship between adolescent anxiety and early adult active smoking. However, a prospective relationship was found in the total sample between active smoking in adolescence and early adult anxiety symptoms (β = 0.17, p<0.05). In the model utilizing data from girls only, a prospective relationship between adolescent active smoking and early adult anxiety symptoms was also found (β = 0.20, p<0.05). Both models showed perfect fit, as expected when all paths are allowed to be estimated freely (chi ^2^ = .000, RMSEA = .000, 90% C.I. RMSEA = .000–.000, TLI 1.00, CFI = 1.00). In both the total sample model and girls only model, level of self-reported anxiety at age 14–15 years was not associated with active smoking at age 18–19 years.. See Figure 1 for both models.

### Smoking as a moderator of the relationship between vulnerability factors in infancy and later anxiety

**Figure 2:**
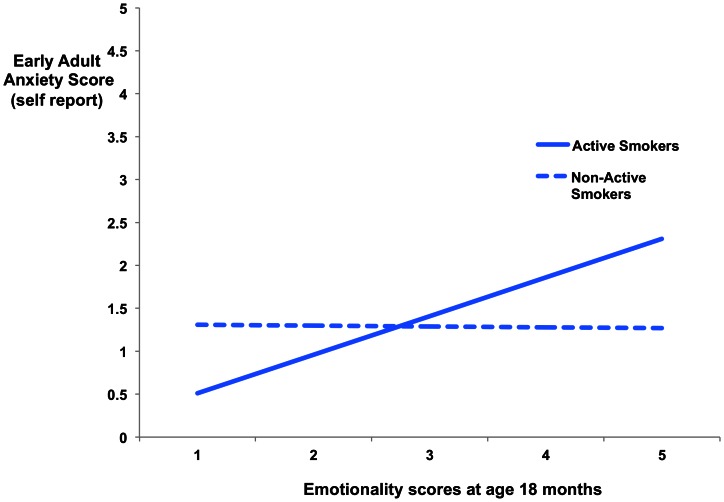
Association between childhood emotionality at 18 months and early adult anxiety scores (18–19 years) by adolescent smoking status. **Caption:** Adolescent active smokers who demonstrated higher emotionality scores during infancy (18months) displayed significantly elevated anxiety in early adulthood that was not present for non-adolescent active smokers.

We tested the moderating effect of adolescent active smoking on the relationship between maternally reported temperament, shyness and internalizing behaviors in infancy and early adult anxiety symptom scores. Initial correlations demonstrated no relationship between infant emotionality (temperament) (r = 0.1, p = 0.83), shyness (r = 0.08, p = 0.12) or internalizing behaviors (r = 0.05, p = 0.28) and early adult anxiety symptoms scores in the total sample. However, freeing equality constraints across smoking groups on the association between infant emotionality and early adult anxiety, significantly improved model fit (Corrected delta chi^2^ = 23.03, df = 1, p<0.01), showing that the association between early vulnerability markers and later anxiety was significantly different among smokers and non-smokers (age 14–15 years). A significant positive association was discovered between infant emotionality and early adult anxiety symptoms scores for active smokers (β = 0.55, p<0.01), that was not present for non-active smokers (β = −0.01, p>0.05) (see Figure 2). This unconstrained model demonstrated excellent fit (chi^2^ = .027, df = 2, RMSEA = .000, 90% C.I. RMSEA = .000–.000, CFI = 1.00, TLI = 1.71). For girls only, freeing the equality constraint across smoking groups lead to a significant improvement of model fit (Corrected delta chi^2^ = 27.01, df = 1, p<0.01), indicating that active and non-active smokers were significantly different in reference to the infant emotionality – early adult anxiety association. A positive association was observed between infant emotionality and early adult anxiety symptoms scores for active smokers (β = 0.52, p<0.01) that was not present for non-active smokers (β = 0.01, p>0.05). Again, the unconstrained model demonstrated excellent fit (chi^2^ = .040, df = 2, RMSEA = .000, 90% C.I. RMSEA = .000–.000, CFI = 1.00, TLI = 4.17).

**Figure 3:**
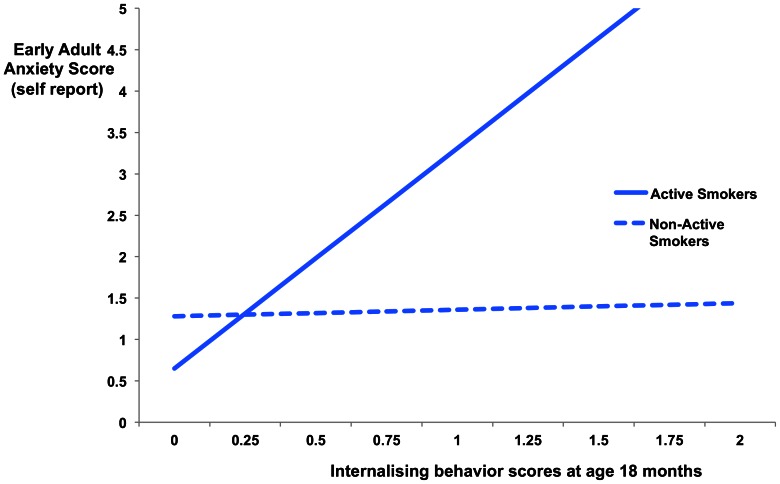
Association between childhood internalizing behavior scores at 18 months and early adult anxiety scores (18–19 years) by adolescent smoking status. **Caption:** Adolescent active smokers who demonstrated higher internalizing behaviors during infancy (18months) displayed significantly elevated anxiety in early adulthood that was not present for non-adolescent active smokers.

Similarly, freeing the equality constraint across smoking groups on the association between infant internalizing behaviors and early adult anxiety symptoms lead to significantly improved model fit (delta Chi^2^ = 14.05, p<0.01, df = 1). In the total sample, this association was significant for active smokers during adolescence (β = 0.85, p<0.01), but was not present for non-active smokers (β = 0.07, p>0.05) (see Figure 3). The fit of the unconstrained model was excellent (chi^2^ = .02, df = 2, RMSEA = .000, 90% C.I. RMSEA = .000–.000, CFI = 1.00, TLI = 1.56). Similar results were found for the girls only model, with model fit improving significantly when freeing the equality constraint across smoking groups (delta Chi^2^ = 11.78, p<0.01, df = 1). The association between infant internalizing symptoms and early adult anxiety was significant for active smokers (β = 0.80, p<0.01), but not for non-active smokers (β = 0.11, p>0.05) during adolescence. Fit of the unconstrained model was excellent (chi^2^ = .03, df = 2, RMSEA = .000, 90% C.I. RMSEA = .000–.000, CFI = 1.00, TLI = 1.49).

**Figure 4:**
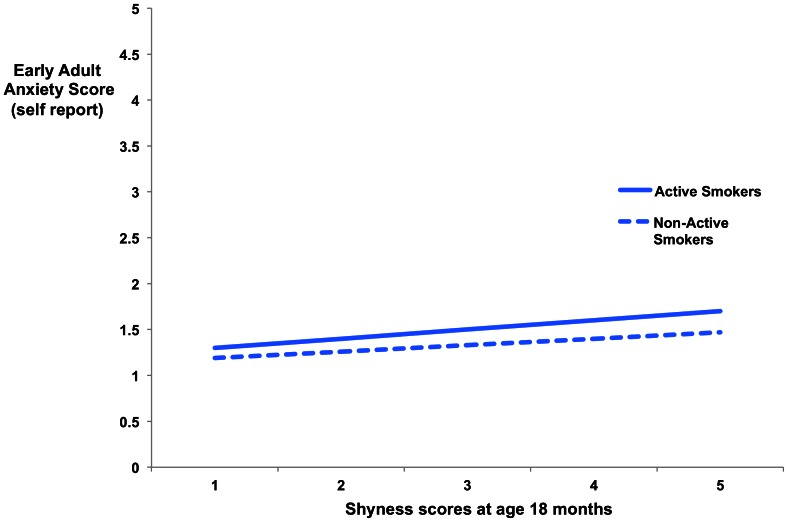
Association between childhood shyness at 18 months and early adult anxiety scores (18–19 years) by adolescent smoking status. **Caption:** No relationship, both for adolescent active smokers and non-active smokers, was discovered between reported infant shyness at 18 months and early adult anxiety scores.

No significant interaction was observed between infant shyness and early adult anxiety symptoms in reference to active smoking (β = 0.08, p>0.05) versus non-active smoking (β = 0.14, p>0.05) (see Figure 4). The constrained model showed excellent fit (chi^2^ = .06, df = 3, RMSEA = .000, 90% C.I. RMSEA = .000–.000, CFI = 1.00, TLI = 1.65.) There was thus no need for a formal test to conclude that freeing the equality constraint across smoking groups would not improve model fit significantly. This was the case for the girls only model as well (active smoking (β = 0.10, p>0.05), non-active smoking (β = 0.15, p>0.05)) (chi^2^ = .06, df = 3, RMSEA = .000, 90% C.I. RMSEA = .000–.000, CFI = 1.00, TLI = 1.00), indicating that smoking did not moderate the shyness-later anxiety relationship. No correlation existed between infant emotionality, internalizing behaviors or shyness and smoking during adolescence.

## Discussion

To our knowledge this is the first study to empirically test the hypothesis that adolescent smoking moderates the relationship between early vulnerability for anxiety and early adult anxiety scores. We found support for this hypothesis in our data. In addition, we demonstrated a prospective relationship between active smoking during adolescence and increased early adult anxiety symptoms, controlling for socioeconomic status.

In our study, both internalizing behaviors and emotionality expressed in infancy were positively associated with early adult anxiety symptoms, but only in those who were active smokers in adolescence. These results suggested that smoking during adolescence conferred a very large effect on the relationship between internalizing behaviors and early adult anxiety symptoms, as well as a moderate to large effect on the relationship between increased emotionality and early adult anxiety symptoms. In contrast, no differences were observed between adolescent smokers and non-smokers in the relationship between infant shyness and early adult anxiety.

There are several potential explanations for the observed moderating effect of active smoking on these relationships. First, it is possible that active smoking during adolescence in this study captured the influence of another factor, or group of factors, that moderate the relationship between these vulnerability behaviors and adult anxiety scores. In this study we controlled for socioeconomic status by utilizing maternal education as a proxy measure, however a number of other potential moderating and confounding factors, including individual (e.g. cognitive style) and social factors (e.g. parental and peer smoking behaviors [60]–[62]), were not available to be included in the models. It will be important for further studies to assess whether such factors affect the relationship between anxiety vulnerability and later anxiety symptoms.

In addition, it is possible that active smoking during adolescence could represent an expected behavior in individuals with an underlying vulnerability for anxiety as part of a developing “anxiety phenotype”. Such a phenotype could progress from infant anxiety vulnerability, through increasing periods of anxiety symptoms, depressive symptoms and psychological distress in late childhood and adolescence that predispose to increased adolescent smoking onset, and finally to increased adult anxiety. Individuals who do not start smoking may overcome this vulnerability phenotype in some way, altering their anxiety trajectory. Numerous investigations have demonstrated “anxiety sensitivity”, the exhibition of fear in response to anxiety-related sensations [63], [64], as being significant in influencing the association between smoking and anxiety symptoms, potentially via increasing anxiety reactions to smoking cues (e.g. nicotine withdrawal) [65], [66].

Another possible explanation is that active smoking is a true risk factor that modulates the translation of infant anxiety vulnerability into early adult anxiety. Numerous potential mechanisms for how cigarette smoking may influence anxiety expression have been postulated. First, much investigation has explored how the effects of smoking on the respiratory and autonomic systems may exacerbate anxiety, particularly panic-type sympto [67]. These outcomes may involve the disease-inducing effects of smoking, negative health perception, and/or acute physiological effects [13], [29], [31]. The primary addictive ingredient in cigarettes, nicotine, is known to increase most measures of physiological arousal, despite often being associated with subjective calming effects [68], and this increased arousal may promote increased anxiety symptoms. Second, an evolving literature suggests exposure to cigarette smoke can adversely affect neurodevelopment. The exposure to cigarette smoke *in utero* has been shown to alter gene expression in areas critical to mood and anxiety control [69], and to modify the expression of infant externalizing behaviors [70] and other behaviors throughout the life course [71]. Adolescence is a critical time for neurodevelopment, characterized by pronounced changes to specific cortical functioning [72], myelination [73] and synaptic connectivity [39], [74]. Components of cigarette smoke including nicotine [39], [75], [76], metals and free radicals [77] can exert deleterious effects on these processes, potentially altering developmental trajectories and leaving individuals more susceptible to increased anxiety symptoms in early adulthood. Although it is unlikely that the above pathways solely explain the large moderating effects demonstrated in the results, our data suggests that further pursuit of this area as one component of an explanatory model for the relationship between anxiety development and cigarette smoking may be warranted.

In contrast to findings for internalizing behaviors and highly emotional temperament, no difference was found between active and non-active smokers in the relationship between infant shyness and early adult anxiety. Temperamental shyness is linked to behavioral inhibition, while emotionality is often linked to approach and pro-active behavior [78], [79]. In line with this, findings demonstrate that shyness is more strongly related to later social anxiety than other forms of anxiety (see Coplan & Rubin [78] for a review). Since our measure of anxiety did not include specific items on social anxiety it's possible that we may have failed to observe a specific relationship in this domain of symptoms in early adulthood. It will be important for future research to examine how smoking moderates the relationship between early vulnerability factors and specific sub-groups of adult anxiety.

Our modeling failed to demonstrate a prospective relationship between increased anxiety symptoms scores at age 14–15 and active smoking age 18–19 years. Data are conflicting [4], [22], [26], [80] on whether anxiety leads to increased smoking behavior [81]. Moderating factors, including peer smoking, family smoking and socioeconomic status, appear important in smoking initiation [16], and further investigation of adolescent smoking initiation in populations with a pre-existing vulnerability to anxiety would be informative.

This study has many strengths but also some limitations. The main strength is the longitudinal follow-up of a large cohort of participants over 18 years. The initial sample of over 900 children was assessed at eight time periods, incorporating self-report data from mothers and children, providing a unique opportunity to model change throughout child development to adulthood. The limitation of missing data that is common in longitudinal studies was managed utilizing the WLSMV estimator in Mplus. Another consideration is the representativeness of the study population. Maternal education was utilized in this study to control for socioeconomic status, as this measure has been strongly linked to health behaviors and conscious lifestyle choices in the Norwegian population [82], although its use is not without contention [83]. However, attrition resulted in the study population becoming progressively skewed, such that the mean level of maternal education increased over time. Given the inverse relationship between socioeconomic status, maternal education and anxiety symptoms, it is possible that both smoking and anxiety symptoms were underrepresented in the sample. Replication of this study in a sample with better coverage of low-SES families would be useful.

We chose *a prior*i to not control for depression in our study, as we believed such analysis would have unacceptably increased the risk of type 2 error. In children and adolescents, anxiety and depressive symptoms are highly comorbid; occurring both sequentially and concurrently [84]. Taking into account the longitudinal nature of our study, controlling for depression would have masked potentially significant individual effects in children who changed between states of anxiety and depression over time. However, to investigate specificity of these results to anxiety, we ran a sensitivity analysis replicating the model with a depression scale and did not find significant effects (data not shown). We therefore conclude that even if shared anxiety/depressive/negative affect symptomatology is responsible for the observed effects, then it is the anxiety component that appears to be more important.

Further, the self-report measure of smoking grouped smokers into frequency groups, as opposed to more definitive criteria such as quantity of cigarettes smoked. It is possible that different reporters would have defined their smoking behavior differently (e.g. occasional vs. daily), potentially affecting the accuracy of smoking rates. We decided to group the relatively small number of participants who identified themselves as “former smokers, now quit” into the non-active smokers category as we felt this most accurately represented their current position. However, we acknowledge that this small group (as former smokers) may have suffered neurodevelopmental effects during their time of smoking that may have altered their anxiety expression, making them similar to active smokers. Although incorrect grouping of these participants may have influenced the results, we feel this effect would be small (due to the small number of participants in this category) and would lead to smaller, not larger, differences between smoking groups. In addition, it is possible that reporters may have been reluctant to report regular smoking in adolescence, lowering reported smoking rates. Our smoking rates were lower than rates of daily smoking reported in a study of Norwegian 15-year-olds in 2005 (8.5% in boys and 9.5% in girls [85]), which may be due to differences in our population (e.g. higher socioeconomic status), under-reporting or a combination of both, with this relatively low sample influencing our ability to undertake more detailed analysis (e.g. of boys only). Nevertheless, under reporting would have acted to attenuate observed relationships, resulting in a more conservative set of findings. Additionally, we were unable to report on rates of nicotine dependence in our study. Individual smokers who are nicotine dependent demonstrate increased levels of anxiety and depression compared to non-dependent smokers [27], and consideration of this will be important in future investigations. In respect to anxiety symptom measures, a different anxiety measurement scale was utilized at age 14–15 and age 18–19. It is possible that this change in symptom scale affected the accuracy in tracking the development of anxiety symptoms over time. However, the fact that anxiety symptoms correlated strongly between time points suggests this is unlikely to have been a significant issue. Finally, our sample did not provide information on other potentially important variables that may influence the relationships between adolescent smoking and anxiety, including parental smoking, parental nicotine dependence, parental anxiety, use of anti-depressant or anti-anxiety medications and more detailed measures of socioeconomic circumstances. Consideration of these factors in future investigations would be prudent.

In conclusion, this study aimed to explore whether smoking may act as an exogenous risk factor that can alter the developmental trajectory of anxiety from infant vulnerability to early adult anxiety symptoms. We discovered a prospective relationship between adolescent active smoking and early adult anxiety, and strong moderating effects of adolescent smoking on the trajectory of some, but not all, measures of anxiety vulnerability in infancy and later adult anxiety symptoms. Although a number of alternative models which themselves are not mutually exclusive may explain these findings, the possibility that smoking is able to exert an adverse effect on the developmental trajectory of anxiety is not discounted by these findings and is worthy of further investigation. Further investigation aimed at confirming this model, coupled with efforts to understand the impact of smoking on adolescent neurodevelopment, may afford an enhanced understanding of how cigarette exposure contributes to anxiety pathogenesis from a developmental perspective. These insights could eventually inform new treatment opportunities (e.g. agents that protect against adverse neurobiological effects) and public health strategies. Although much remains unclear, given the known adverse health effects of cigarette smoking, in conjunction with the significant health burden imposed by anxiety disorders [86], [87], this study does support the importance of smoking prevention and cessation programs targeting children and adolescence. Smoking should be considered as one of the potentially plastic environmental risk factors for common mental disorders, and needs to be incorporated into prevention and promotion efforts [88]. These efforts may exert positive effects not just on characteristics of physical health but also on anxiety.
